# Blood Pressure and Transient Postoperative Neurologic Deterioration, Following Superficial Temporal-to-Middle Cerebral Artery Anastomosis in Adult Patients with Moyamoya Disease: A Retrospective Cohort Study

**DOI:** 10.3390/jcm10122567

**Published:** 2021-06-10

**Authors:** Tak-Kyu Oh, Ji-Hyeon Kim, Ho-Young Lee, Seong-Eun Kim, Tac-Keun Kim, Jae-Seung Bang, Moon-Ku Han, Chang-Wan Oh, Hee-Joon Bae, Young-Tae Jeon

**Affiliations:** 1 Department of Anesthesiology and Pain Medicine, Seoul National University Bundang Hospital, Seoul National University College of Medicine, Seongnam 13620, Korea; airohtak@hotmail.com; 2 Department of Nuclear Medicine, Seoul National University Bundang Hospital, Seoul National University College of Medicine, Seongnam 13620, Korea; jhleesnubh@gmail.com (J.-H.K.); debobkr@snubh.org (H.-Y.L.); 3 Department of Neurology and Cerebrovascular Center, Seoul National University Bundang Hospital, Seoul National University College of Medicine, Seongnam 13620, Korea; sekim1004@gmail.com (S.-E.K.); bluethink4@snubh.org (M.-K.H.); 4 Department of Neurosurgery, Seoul National University Bundang Hospital, Seoul National University College of Medicine, Seongnam 13620, Korea; nskim@snubh.org (T.-K.K.); nsbang@snubh.org (J.-S.B.); wanoh@snubh.org (C.-W.O.)

**Keywords:** neurosurgery, blood pressure, neurologic manifestations, general surgery, postoperative complications

## Abstract

We investigated whether intraoperative systolic blood pressure (ISBP) is associated with the risk of transient neurologic deficits (TND) following superficial temporal-to-middle cerebral artery (STA-MCA) anastomosis in adult patients with moyamoya disease (MMD). In this retrospective observational study, data from adult patients with MMD who had undergone STA-MCA anastomosis at a single tertiary academic hospital during May 2003–April 2014 were examined. Data on patient characteristics were obtained from electronic medical records, including the details of comorbidities and laboratory findings. TND was the primary outcome of interest. Out of 192 patients (228 hemispheres), 66 (29%) hemispheres had TND after surgery. There were significant differences in ISBP between patients with and without TND. The lowest ISBP quartile was independently associated with TND (odds ratio: 5.50; 95% confidence interval: 1.96–15.46). Low ISBP might lead to TND after STA-MCA anastomosis in adult patients with MMD. In patients with poor perfusion status, low ISBP was associated with an increased risk of TND. Our findings suggest that strict ISBP control might be required to prevent TND after anastomosis in patients with MMD, in particular, in patients with poor perfusion status. Given limitations due to the retrospective design, further studies are needed to clarify these findings.

## 1. Introduction

Superficial temporal-to-middle cerebral artery (STA-MCA) anastomosis is a surgical treatment to reduce the risk of cerebral ischemia in patients with moyamoya disease (MMD) [1]. Transient neurological deficits (TND) can occur due to cerebral hyper- [2,3,4] or hypoperfusion associated with this procedure [5]. Previous studies have reported several risk factors for TND, including adult onset, hemorrhagic onset [6], surgery to the dominant hemisphere, and increased postoperative white blood cell counts [7].

Blood pressure (BP) variability has been associated with neurologic deterioration following acute ischemic stroke [8]. A common practice in MMD management is to keep BP at or just above the preoperative baseline [9]. A decline in the mean arterial pressure during surgery has been reported to cause cerebral ischemia by decreasing cerebral perfusion pressure and cerebral blood flow in childhood MMD patients receiving revascularization surgery [10]. In pediatric MMD patients undergoing STA-MCA anastomosis, decreased reserve capacity in postoperative SPECT was associated with neurological deficit and ischemic attacks [11]. Li et al. reported that a higher variance of intraoperative BP and drastic BP decline were independent risk factors for the development of postoperative infarction in MMD patients who underwent revascularization surgery [12]. In addition, Jung et al. reported that hypotension was associated with an increased risk of ischemic stroke after surgical treatment in patients with MMD. However, these studies did not focus on the impact of perioperative BP on the risk of TND in these patients.

Therefore, we aimed to elucidate whether intraoperative BP status is associated with the risk of TND following STA-MCA anastomosis in adult patients with MMD, and whether preoperative cerebral perfusion status might mediate this association.

## 2. Materials and Methods

### 2.1. Design and Ethical Statement

This retrospective observational study was approved by the institutional review board of Seoul National University Bundang Hospital (IRB No.B-1411/274-113). The requirement for patient consent was waived due to the retrospective nature of this study and minimal risk to study participants. Data were extracted from electronic medical records.

### 2.2. Study Subjects and Data Collection

We collected data on patients with adult MMD who had undergone STA-MCA anastomosis at Seoul National University Bundang Hospital between May 2003 and April 2014. Two hundred forty-eight hemispheres of 208 consecutive patients met the inclusion criteria; however, 20 hemispheres were subsequently excluded due to insufficient data (8), postoperative cerebral hemorrhage (6), and postoperative cerebral infarction (6), leaving 228 hemispheres in the final sample.

Patient data were extracted from electronic medical records, including patient characteristics, details of comorbidities, and laboratory findings. BP data were collected through routine clinical practice and downloaded from our institution’s clinical data warehouse for the purpose of this study.

### 2.3. Surgical and Anaesthetic Procedures

General indication for revascularization surgery included apparent cerebral ischemia or decreased regional blood flow, vascular response, and reserve in perfusion studies in our hospital [13]. Digital subtraction angiography was performed to diagnose MMD, evaluating collateral channels and identifying appropriate donor and recipient. Thereafter, perfusion status was evaluated with computed tomography (CT) or single-photon emission computed tomography (SPECT) with acetazolamide challenge, useful for evaluating resting perfusion and reserve capacity.

Regarding anesthetic techniques, similar protocols were followed with study participants. Radial artery cannulation was performed to perioperatively monitor blood pressure. Total intravenous anesthesia with propofol and remifentanil was administered with a target-controlled infusion pump. Intraoperative systolic BP (ISBP) was maintained above the level of the preoperative baseline value. Normocapnia and normovolemia were maintained.

After surgery, brain CT was performed to detect any postoperative infarction or hemorrhage. Patients were admitted to a neurological intensive care unit. Neurological examination was performed once patients regained consciousness. Systolic BP (SBP) was kept within 20 mmHg of the preoperative level. Patients’ volume status was maintained as euvolemic.

### 2.4. Brain Perfusion Diamox SPECT Image Analysis

Two nuclear medicine physicians (HYL and JHK) performed independent visual assessments of the SPECT images of each cerebral hemisphere. Disagreements were resolved by discussion and consensus between the physicians. Perfusion status detected on brain SPECT images was classified as “normal”, “mild decreased”, “moderate decreased”, “severe decreased”, or “defect”. Decreased perfusion was defined as cerebral perfusion on basal SPECT within a lower color range compared with contralateral side and/or the surrounding area. Decreased cerebrovascular reserve (CVR) was defined as abnormal cerebral perfusion within a lower color range relative to basal SPECT, after acetazolamide administration. The severity of decreased CVR was classified into “mild”, “moderate”, “severe”, and “defect” [14]. We evaluated the concordance rate between the physicians’ evaluation, which was 89.5%.

### 2.5. Outcome Assessment: TND

The primary outcome of interest was the occurrence of TND after surgery. Based on a previous report [3], TND was confirmed when the patient met all the following four criteria: (1) presence of neurological deficits that did not exist before surgery, and the deficit including both ipsilateral and contralateral symptoms; (2) TND resolved completely within 15 days of operation; (3) presence of a significant focal increase (hyperperfusion) or decrease (hypoperfusion) in cerebral blood flow at the anastomosis site, captured by a postoperative SPECT scan; (4) no hematoma or acute infarction detected on brain CT or diffusion-weighted magnetic resonance imaging. If a case met 1–3 criteria from among the four criteria above, TND was not indicated in this study. If neurological symptoms developed after surgery, SPECT or perfusion CT was performed to evaluate changes in cerebral perfusion status.

### 2.6. Confounders

The following variables were collected and used as confounders in this study: age, sex, mean preoperative SBP, operation time, operation site (right/left), history of smoking, preoperative hemoglobin level, and white blood cell count. In addition, some comorbidities were used as confounders, such as previous stroke, hypertension, diabetes mellitus, coronary artery disease, and thyroid disease. The data on preoperative SBP were collected using the mean value of all SBP readings from hospital admission to entry into the operating room.

### 2.7. Statistical Analysis

ISBP data were summarized as a mean, categorized into four groups, using quartiles, and examined for an association with potentially confounding variables. One-way ANOVA or Kruskal–Wallis test was conducted for continuous variables, and chi-square test or Fisher’s exact test was conducted for categorical variables.

According to TND, the categorized mean ISBP was compared using the chi-square test or Fisher’s exact test. Proportion of confirmed TND cases per mean ISBP category was plotted against the patients’ cerebral perfusion status. The association between TND and mean ISBP was examined by comparing odds ratios (ORs) in a logistic regression model. ORs were adjusted for confounding variables in a multivariable model. Variables with a *p*-value less than 0.1 in the univariable analysis were included in the multivariable model for adjustment. The following variables were included in the multivariable model: age, cerebral perfusion status, operation duration, operation site, history of stroke, duration of hospitalization, pre-operation mean SBP, preoperative hemoglobin level, and postoperative hemoglobin level.

In the multivariable logistic regression model, a nonlinear term representing the mean ISBP was included to examine a nonlinear relationship between mean ISBP and TND risk. To control for multicollinearity, cantered mean ISBP was used. Moreover, cerebral perfusion status was included as an interaction term to verify its impact on the association between mean ISBP and TND risk.

Finally, the proportion of confirmed TND cases per ISBP category predicted by the multivariable regression model was plotted per mean ISBP according to the cerebral perfusion status, controlling for relevant confounders, whereby averages and reference values were used for continuous and categorical variables, respectively. Statistical analyses were performed using SAS 9.4 (SAS Institute Inc., Cary, NC, USA). *p*-values < 0.05 were considered statistically significant.

## 3. Results

Among 228 hemispheres of 192 patients, 66 hemispheres (29%) had confirmed TND after surgery. Among these patients, 30,853 ISBP measurements were taken, with an average of 136 measurements per patient. Mean (standard deviation, SD) of ISBP per patient was 129.6 (10.9) and 14.8 (4.0). There were no significant differences in baseline characteristics among the groups based on ISBP quartiles (Table 1). TND was significantly associated with mean ISBP (*p* = 0.005, Table 2). Using ISBP values within 125–133 mmHg (second and third quartile, respectively) as a reference, the odds of TND in patients with mean ISBP < 120.2 mmHg were 5–6 times greater than in the reference group (crude OR = 4.78, 95% CI: 1.92–11.91; adjusted OR = 5.50, 95% CI: 1.96–15.46). Meanwhile, for patients with mean ISBP in the 125–133 mmHg range, the odds of TND were three times greater than for the reference group (crude OR = 2.83, 95% CI: 1.11–7.19; adjusted OR = 3.24, 95% CI: 1.16–9.05) (Table 2).

**Figure 1:**
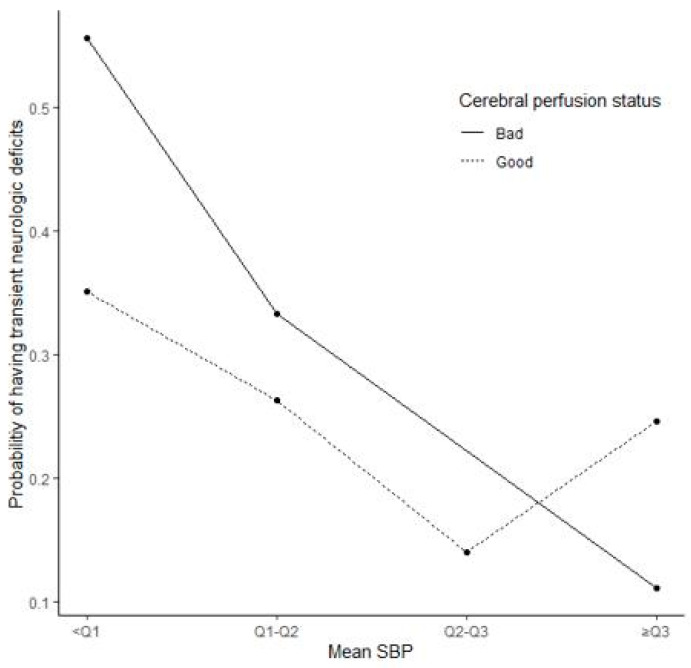
Proportion of transient neurologic deficits across quartile group of mean systolic blood pressure (SBP). Poor perfusion was defined as cerebral perfusion within a lower color range on basal SPECT compared with contralateral side and/or the surrounding area, while good perfusion status detected on brain SPECT images was classified as “normal” according to visual assessment by two nuclear medicine physicians.

**Figure 2:**
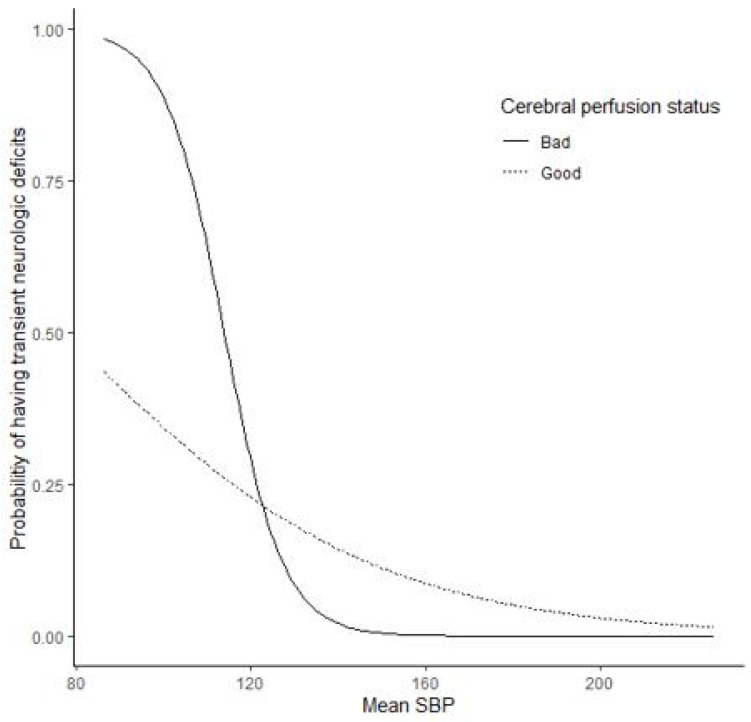
Predicted probabilities of transient neurologic deficits derived from a linear logistic regression model on mean systolic blood pressure (SBP). Poor perfusion was defined as cerebral perfusion within a lower color range on basal SPECT compared with contralateral side and/or the surrounding area, while good perfusion status detected on brain SPECT images was classified as “normal” according to visual assessment by two nuclear medicine physicians.

The impact of mean ISBP on TND risk was mediated by the cerebral perfusion status (Figure 1). However, the quadratic term and interaction test result were nonsignificant (*p* = 0.355 and *p* = 0.157, respectively).The mean ISBP had an effect on the proportion of TND cases (*p*-value = 0.026), which was independent of the patients’ cerebral perfusion status (*p*-value = 0.123). The risk of TND decreased as the mean ISBP increased. In addition, preoperative mean SBP, operation site, preoperative hemoglobin level, and hospital stay duration were associated with the proportion of TND cases confirmed within each mean ISBP category. Overall, in each mean ISBP group, patients with a higher mean preoperative SBP or preoperative hemoglobin level, longer hospital stay, and patients operated on the left side were more likely to develop TND (Table 3). As observed from the trends of Figure 1 and Figure 2, the mean ISBP can be nonlinear, and the curve can vary according to the cerebral perfusion status. Therefore, nonlinear logistic regression models with or without interaction with perfusion status were constructed in addition to a linear logistic regression model.

As observed from the trends of Figure 1 and Figure 2, the mean ISBP can be nonlinear, and the curve can vary according to the cerebral perfusion status. Therefore, nonlinear logistic regression models with or without interaction with perfusion status were constructed in addition to a linear logistic regression model.

## 4. Discussion

This study has shown that low ISBP was independently associated with the risk of postoperative TND following STA-MCA anastomosis in adult patients with MMD. In patients with a poor perfusion status, low ISBP further increased the risk of TND.

The association between intraoperative SBP and risk of stroke has been reported in patients undergoing cardiac [15] or general surgery [16]. These previous studies emphasized the importance of preventing intraoperative hypotension for maintaining cerebral perfusion during surgery. Consistent with these studies, our findings suggest that the risk of TND after revascularization surgery for MMD might be associated with low ISBP, which might cause local intraoperative hypoperfusion. A recent study demonstrated that local hypoperfusion measured with cerebral blood flow was correlated with transient neurologic events [17]. A variety of factors affect low blood pressure during surgery, including anesthetics. The anesthetic agents commonly used for neurosurgery, such as propofol and remifentanil, can lower blood pressure. In the present study, phenylephrine was used to maintain BP above the preoperative baseline value. Although the effect of phenylephrine has not been studied in patients with MMD, administration of vasopressors to patients with ischemic stroke has been associated with improvement in neurologic outcomes [18].

In the present study, cerebrovascular reserve was associated with the risk of TND. Poor preoperative perfusion status increased the risk of TND when ISBP decreased. Most ischemic events in patients with MMD have been ascribed to reduced blood flow caused by the narrowing of cerebral arteries [19]. Previously, an examination with postoperative perfusion SPECT imaging revealed that patients with a decreased cerebrovascular reserve are at risk of neurologic deficits and ischemic attacks [11]. The present study emphasizes the role of perfusion status alongside changes to blood pressure in managing these outcomes.

Operation to the left side is a well-documented risk factor for hyperperfusion after STA-MCA anastomosis [7,20]. Similarly, operation to the left side was associated with the risk of TND in the present study. These findings suggest that surgery to the left side requires careful management, including maintenance of adequate cerebral flow during surgery, which might require more fine-tuning compared to when operating on the right side.

### Limitations

The present study has some limitations. First, selection bias was inevitable due to the retrospective nature of this study. Second, our data analysis was performed per hemisphere rather than per patient, meaning we were not able to account for cases of patients who had undergone surgery on both sides (right and left). We were not able to discount the possibility that double surgery might affect outcomes. Third, factors related to the type of surgery (e.g., direct or combined bypass) were not accounted for in the analysis. Fourth, we divided the mean ISBP into four groups as categorical variables for statistical analysis, and Q2–Q3 (125–133) was used as a reference. However, controlling and maintaining SBP during STA-MCA anastomosis among MMD patients might be difficult in clinical practice. Therefore, SBP of 125–133 should be considered as the target; our results showed that avoiding lower SBP during STA-MCA anastomosis among MMD patients was associated with decreased TND. Fifth, we did not consider postoperative SBP in this study, which may affect the development of TND during the postoperative period. Lastly, we did not consider preoperative ischemic symptoms in this study. As preoperative ischemic symptoms are independent risk factors for postoperative ischemia among MMD patients [21], they also may have affected our results.

## 5. Conclusions

In summary, this retrospective cohort study demonstrated that low ISBP might be associated with TND risk following STA-MCA anastomosis in adult patients with MMD. In patients with poor perfusion status, low ISBP increased the risk of TND. Our findings suggest that strict ISBP control might be required to prevent TND in this patient group, in particular, in patients with a poor perfusion status. Given the limitation due to retrospective design, further studies are needed to clarify these findings.

## Figures and Tables

**Table 1:** Comparison of baseline characteristics by SBP mean quartiles.

Variable	<Q1 (<120.2) (*n* = 57)	Q1–Q2 (120.2–125) (*n* = 57)	Q2–Q3 (125–133) (*n* = 57)	≥Q4 (≥133) (*n* = 57)	*p*-Value *
Age (year)	38.9 (13.2)	39.0 (12.4)	41.5 (12.0)	41.7 (12.2)	0.471
Gender: Male	20 (23)	25 (29)	17 (20)	24 (28)	0.382
BMI (kg/m^2^)	25.2 (4.2)	24.8 (4.0)	26.6 (4.3)	26 (4.3)	0.129
Comorbidities					
Previous Stroke	9 (32)	5 (18)	6 (21)	8 (29)	0.653
Hypertension	16 (21)	17 (23)	22 (29)	20 (27)	0.613
Diabete Mellitus	6 (18)	12 (35)	9 (26)	7 (21)	0.407
Smoking	13 (27)	16 (33)	8 (16)	12 (24)	0.333
Coronary Artery Disease	0	0	2 (100)	0	0.247 ^§^
Thyroid Disease	4 (29)	4 (29)	3 (21)	3 (21)	1.000 ^§^
Clinical Characteristics					
Anesthesia Time (min)	479 (91)	474 (100)	499 (102)	492 (95)	0.487
Operation Time (min)	408 (86)	406 (108)	423 (99)	424 (93)	0.642
Preop Hb (g/dL)	13.8 (1.6)	13.8 (1.7)	13.5 (1.6)	13.5 (1.6)	0.451
Preop WBC (10^3^/uL)	7.0 (1.9)	7.0 (1.7)	6.7 (1.4)	6.7 (1.5)	0.477
LOS (day)	13.4 (4.7)	12.5 (4.4)	12.4 (4.4)	14.8 (7.5)	0.07
Operation site (Rt/Lt)	31 (24)/26 (27)	33 (25)/24 (24)	34 (26)/23 (23)	32 (25)/25 (26)	0.949
Cerebral Perfusion status	8 (30.8)	5 (19.2)	4 (15.4)	9 (34.6)	0.399
Preop mean SBP	113.8 (13.2)	118.5 (15.1)	127.5 (16.2)	128.5 (15.2)	0.128

Values are expressed as the mean (standard deviation) or number (percentage, %). * By one-way ANOVA or chi-square test as appropriate. ^§^ By Kruskal–Wallis test or Fisher’s exact test as appropriate. SBP, systolic blood pressure; BMI, body mass index; Preop, preoperative; LOS, length of hospital stay; Rt, right; Lt, left; Hb, hemoglobin; WBC, white blood cells.

**Table 2:** Occurrence of TND according to ISBP quartiles.

ISBP Quartiles	TND (+) (*n* = 66)	TND (−) (*n* = 162)	*p* Value *	Crude OR (95% CI) ^§^	Adjusted OR (95% CI) ^†^
<Q1 (<120.2)	25 (43.9%)	32 (56.1%)	0.005	4.78 (1.92, 11.91)	5.50 (1.96, 15.46)
Q1–Q2 (120.2–125)	18 (31.6%)	39 (68.4%)		2.83 (1.11, 7.19)	3.24 (1.16, 9.05)
Q2–Q3 (125–133)	8 (14.0%)	49 (86.0%)		reference	reference
≥Q3 (≥133)	15 (26.3%)	42 (73.7%)		2.19 (0.84, 5.67)	1.39 (0.48, 4.04)

* By chi-square test as appropriate or Fisher’s exact test as appropriate. ^§^ By logistic regression model; modeling the probability of TND. ^†^ By logistic regression model; modeling the probability of TND adjusted for age, operation time, operation site, cerebral perfusion status, history of stroke, duration of hospitalization, pre operation SBP mean, preoperative hemoglobin, and postoperative hemoglobin. TND, transient neurologic deterioration; OR, odds ratio; ISBP, intraoperative systolic blood pressure.

**Table 3:** Estimates from the multivariable logistic regression models on ISBP mean.

Model	Linear		Nonlinear without Interaction		Nonlinear with Interaction	
Parameter	Estimate	*p*-Value	Estimate	*p*-Value	Estimate	*p*-Value
ISBP mean	−0.0883	0.026	–	–	–	–
Cerebral perfusion status	7.3463	0.124	−0.0558	0.833	−0.2131	0.512
ISBP mean * cerebral perfusion status	−0.0599	0.123	–	–	–	–
Centered ISBP mean			−0.0470	0.01	−0.0918	0.018
Quadratic term of mean			0.0009	0.355	0.0009	0.38
Centered ISBP mean * cerebral perfusion status			–	–	−0.0538	0.157
Age	−0.0280	0.052	−0.0288	0.046	−0.0281	0.053
Preoperative SBP mean	0.0149	0.003	0.0439	0.003	0.0414	0.006
Operation time	−0.00126	0.484	−0.0012	0.505	−0.0012	0.502
Operation site: left side	0.3907	0.022	0.3809	0.025	0.3849	0.024
Previous stroke	0.0450	0.855	0.0787	0.749	0.0589	0.812
Preoperative Hemoglobin	0.3866	0.005	0.3805	0.006	0.3730	0.007
Postoperative Hemoglobin	−0.2010	0.156	−0.1746	0.219	−0.1774	0.213
Length of hospital stay	0.1089	0.002	0.0966	0.004	0.1021	0.004

* Interaction between the two variables.SBP, systolic blood pressure. Reference group: bad cerebral perfusion status, right site of operation, and no previous stroke.

## Data Availability

Data will be available upon reasonable request to corresponding authors.
